# Associations of dietary inflammatory index scores and serum inflammatory factors with the risk of osteoporosis: a cross-sectional study from Xinjiang, China

**DOI:** 10.1186/s13018-024-04866-x

**Published:** 2024-07-23

**Authors:** Min Tong, Huanwen Zhang, Yuanyuan Li, Wenhui Fu, Tao Luo, Jianghong Dai, Yifei Huang

**Affiliations:** 1 Spine Division 2, Traditional Chinese Medicine Hospital of Xinjiang Medical University, Urumqi, 830000 China; 2 School of Public Health, Xinjiang Medical University, 567 Shangde North Road, Urumqi, 830000 China; 3 Center for Disease Control and Prevention of Xinjiang Uygur Autonomous Region, Urumqi, 830011 China

**Keywords:** Dietary inflammatory index, Inflammation, Osteoporosis, Epidemiology

## Abstract

**Background:**

Previous studies have shown that the inflammatory potential of the diet is associated with a variety of chronic noncommunicable diseases characterized by a chronic low-grade inflammatory response. However, the relationships between dietary inflammatory potential and organismal inflammatory status and osteoporosis have been less studied. This study aimed to investigate the relationships among inflammatory diet, inflammatory state and osteoporosis in the Xinjiang multiethnic population.

**Methods:**

The participants consisted of 4452 adults aged 35 to 74 years from Xinjiang, China. The dietary inflammatory index (DII) was calculated using dietary data collected with a semiquantitative food frequency questionnaire, and information about osteoporosis was derived from quantitative ultrasound measurements. The relationships of the DII score and inflammatory factors with the risk of osteoporosis were analysed using multivariate logistic regression, and the nonlinear associations between DII and osteoporosis were further analysed using restricted cubic splines.

**Results:**

The results showed that proinflammatory diets were associated with a greater risk of osteoporosis (T3 vs. T1: *OR* = 1.87; 95% *CI* = 1.44, 2.45) and that there was no nonlinear relationship between the DII and the risk of osteoporosis. Increased concentrations of the inflammatory factors IL-6, IL-10, IL-12p70, IL-17, and IL-23 were associated with a greater risk of osteoporosis.

**Conclusions:**

The risk of osteoporosis can be reduced by increasing the consumption of an appropriate anti-inflammatory diet.

**Supplementary Information:**

The online version contains supplementary material available at 10.1186/s13018-024-04866-x.

## Background

Osteoporosis is a systemic metabolic disease and a common risk factor for fractures, which can lead to a generalised reduction in bone mass and microstructural damage to bone tissue through imbalances in bone metabolism, which in turn leads to increased bone fragility and increased fracture risk [1]. It can occur at any age, has a serious impact on people’s quality of life and results in a heavy economic burden. The most serious consequence of osteoporosis is fracture, occurring most often in the hip, vertebrae and distal radius, with a mortality rate of nearly 20% in the first year after hip fracture [2]. The annual cost of treating osteoporosis-related fractures in the United States can reach $17 to $20 billion, exceeding even the medical costs of myocardial infarction, breast cancer, and cerebrovascular accidents [3]. In China, by 2035, the number of osteoporosis-related fractures is expected to reach 4.83 million cases per year, at an annual cost of approximately $19.92 billion [4]. Therefore, the identification of risk factors associated with osteoporosis is particularly important for reducing the occurrence of fractures and the associated health care costs.

Chronic inflammation has emerged as a potential risk factor for a variety of noncommunicable diseases, including obesity, type 2 diabetes, cardiometabolic diseases, cancer, and depression [5–7]. Chronic inflammation is also gaining attention as a potential risk factor for osteoporosis [8]. Several population and experimental research studies have shown that serum inflammatory factors play an important role in the development of osteoporosis [7–10]. Studies in premenopausal and postmenopausal women revealed that elevated serum CRP levels were associated with lower bone mineral density (BMD) and a greater risk of osteoporosis [9]. Experimental studies have shown that the serum inflammatory factors IL-1 and TNF-α produced by macrophages and monocytes stimulate osteoclast differentiation [10], which in turn increases the number of osteoclasts and bone resorption, participates in the development of osteoporosis, and can lead to increased bone fragility and fracture risk [8, 11].

Foods, nutrients and bioactive substances are important regulators of inflammation in organisms. For example, red meat, refined grains and saturated fatty acids have significant proinflammatory potential and are associated with increased levels of inflammatory factors such as CRP and IL-6 [12, 13], whereas fruits, vegetables, legumes, omega-3 fatty acids and omega-6 fatty acids have significant anti-inflammatory potential and are associated with decreased levels of inflammatory factors [14, 15]. Individual study participants do not consume only one food/nutrient in isolation but rather consume a combined dietary spectrum of various foods, and the dietary inflammatory index (DII) can be used to assess the overall dietary inflammatory response potential of study participants by analysing the level of inflammatory response to different dietary components [16]. The DII is a dietary inflammation scoring system based on the effects of food/nutrients on six inflammatory markers of the body, namely, CRP, TNF-α, IL-6, IL-1β, IL-4, and IL-10, according to previous literature, with higher DII scores indicating greater proinflammatory potential [17]. Some studies have confirmed that higher DII scores are associated with higher levels of inflammatory factors [18, 19], suggesting that the DII may help to elucidate the relationship between inflammatory responses and chronic noncommunicable diseases.

It has been shown that the DII is significantly associated with a variety of chronic noncommunicable diseases characterized by chronic low-grade inflammation [5–8], but the relationship between dietary inflammatory potential and the inflammatory status of the organism and osteoporosis has been less studied. Xinjiang is a region with people of multiple races, and different race groups have unique dietary structures. In this study, we aimed to examine the relationships among inflammatory diet, organismal inflammatory status and osteoporosis in different racial groups in Xinjiang Province with distinctive dietary characteristics and to provide a scientific basis for studying the inflammatory mechanisms of osteoporosis.

## Methods

### Study population and data collection

**Fig. 1:**
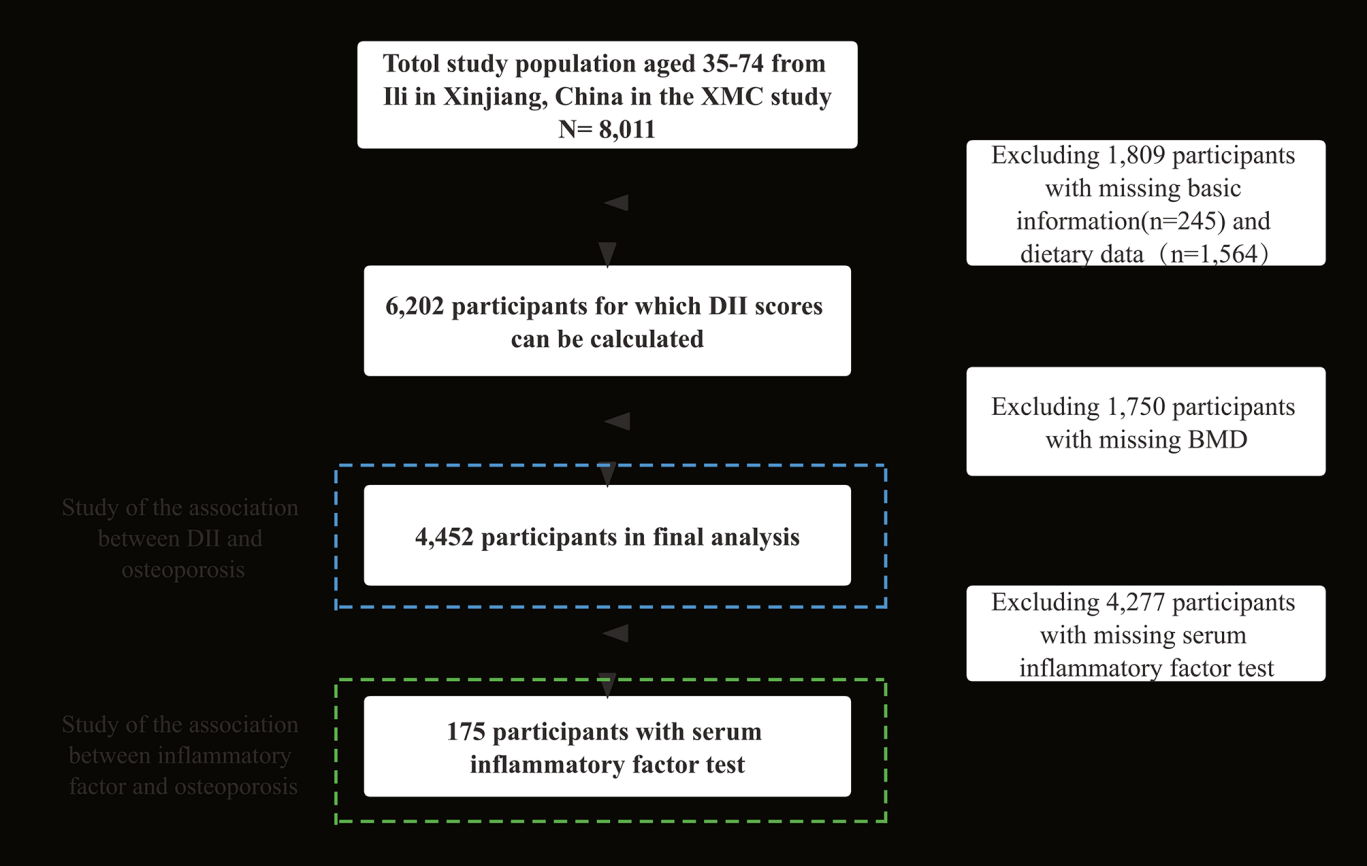
Flow chart of the analysis of the samples in the present study

The XMC study was a prospective cohort study involving participants from Urumqi, Ili, and Hotan in Xinjiang, China. The XMC study investigated the potential causal relationships of factors such as dietary behaviour habits and lifestyle factors with health outcomes in the Xinjiang population. Our study analysed baseline survey data from Ili, which included 8011 residents aged 35–74 years. The exclusion criteria for this study were (1) residents who were out of the age range in the actual survey but volunteered to participate in the survey; (2) residents who lacked basic information, BMD, and dietary data in the baseline survey; and (3) residents who lacked height and weight data in the baseline physical examination. Finally, 4171 participants were included in this study for statistical analysis (Fig. 1). This study was approved by the ethics committee before the start of the survey (2018XE0108), and all study participants signed the informed consent form. Detailed information on the XMC study has been published previously [20].

### Dietary assessment using the FFQ and the calculation of the DII

Based on the unified questionnaire of the XMC project, the final dietary questionnaire included 127 food items, taking into account the dietary characteristics of Xinjiang. The frequency (daily, 4–6 times/week, 1–3 times/week, 1–3 times/month, no food or very little food) and the amount of each food consumed by the study participants in the past year were investigated and converted into daily intakes.

The calculation of DII was designed and developed by Shivappa et al. [17] in 2014, and the algorithm is based on a review of 1,943 articles linking 45 food parameters to 6 inflammatory biomarkers (IL-1β, IL-4, IL-6, IL-10, TNF-α and C-reactive protein). The detailed calculation steps were as follows: (1) dietary intake data were obtained from the study participants through dietary surveys; (2) dietary intake data were converted into food components included in the DII according to the 2016 version of the Chinese food composition table; (3) dietary intake data of the study participants were compared with the global standard dietary intake database, and the mean and standard deviation of each nutrient intake were calculated; (4) the specific inflammatory potential score for each nutrient was obtained by reviewing the literature [17]; (5) the “individual-specific DII score” for each nutrient was calculated (individual-specific DII score = *Z* × specific inflammatory effect score); and (6) the “individual-specific DII scores” for all nutrients in the study were summed to obtain the “individual” DII score. The higher the DII is, the stronger the proinflammatory effect of the diet, and the greater the negative value is, the stronger the anti-inflammatory effect of the diet. Since the Chinese food composition table is not fully harmonized with that of the United States, the DII was calculated based on the content of 24 nutrients in our study; however, previous studies have confirmed that DII scores based on < 30 nutrients are still reliable [17, 21].

### BMD measurement and osteoporosis diagnosis

We measured BMD at the heel using a SONOS-T2000 quantitative ultrasound bone sonometer (QUS) provided by OSTO. The subjects were told to take off their shoes and socks, apply a coupling agent to the front of the QUS probe, and place the probe at the heel for measurement. Osteoporosis was diagnosed based on QUS measurements with a T value≤-2.5 [22].

### Serum inflammatory factor test

Thirteen indicators, IL-1β, TNF-α, IL-6, IL-10, IFN-α, IFN-γ, IL-8, MCP-1, IL-12p70, IL-17, IL-18, IL-23, and IL-33, were measured using a multicomponent flow analysis kit produced by BioLegend Biologics Ltd.

### Covariates

Basic information (including information on sex, age, ethnicity, education level, and marital status), dietary information, lifestyle information (smoking status, alcohol consumption status, and physical activity), and disease history (history of fracture) of the study participants were collected by a uniformly trained investigator via a face-to-face survey through a questionnaire. Age was measured as a continuous variable, race was divided into five categories—Han, Hui, Uyghur, Kazakh and other ethnic groups—and educational level was divided into three categories—elementary school and below, middle school and high school and above. Alcohol consumption, smoking status and physical activity status were divided into three categories based on frequency: frequently, occasionally and never. In addition, disease history information was determined by questionnaires or diagnostic certificates from local hospitals. Height, weight and other physical examination indices are examined by doctors and technicians who have obtained relevant qualifications and experience using uniform standards and uniform specifications of equipment for operation. Body mass index (BMI) was calculated as BMI = weight (kg)/height^2^ (m^2^).

### Statistical analysis

To analyse the association between DII scores and the risk of osteoporosis, the DII scores were divided into tertiles. We used the mean (standard deviation) to present continuous data satisfying a normal distribution, the median (interquartile range) to present continuous data not satisfying a normal distribution, and the frequency (proportion) to present categorical variables. We applied ANOVA for normally distributed continuous data for comparison, the Kruskal‒Wallis test for nonnormally distributed continuous data for comparison, and the chi-square test for categorical data. Logistic regression models were used to estimate the associations of the DII score and inflammatory factors with the risk of osteoporosis. In addition, the nonlinear association between DII and osteoporosis was further explored using restricted cubic splines. In the main analysis, the multivariate model was adjusted for sex, age, race, BMI, education level, alcohol consumption, smoking status, physical activity, and history of fractures. In the sensitivity analysis, energy intake was added to the multivariate model as a covariate for adjustment. The test level was α = 0.05, and a two-sided *P* value of < 0.05 was considered to indicate statistical significance. All the statistical analyses were performed using R 4.2.3.

## Results

### Characteristics of study participants

A total of 4171 study participants who underwent 175 tests for inflammatory factors were enrolled in the final analysis. The mean age of the study participants was 50.26 years, and participants with higher DII scores were older. A total of 53.92% of the participants were female, approximately 33.45% of the participants were Uyghurs, 71.57% of the participants had an education level of elementary school or less, and there were significant differences in the distribution of race and education level between the DII tertiles (*P* < 0.05). Approximately 22.46% of the participants were frequent smokers, 0.86% of the participants were frequent drinkers, and 4.58% of the participants were frequent physical activity participants, with the number of frequent smokers, drinkers, and physical activity participants significantly decreasing in the highest DII tertiles (*P* < 0.05). See Table 1 for details.

**Table 1:** The basic characteristics of participants by dietary inflammatory index (DII) tertiles

Characteristic	All participants	Tertiles of Dietary Inflammatory Index (DII)			*P* value
		T1	T2	T3	
*N*	*n* = 4,171	*n* = 1,377	*n* = 1,417	*n* = 1,377	
DII (range)	-6.9732 ∼ 4.3006	-6.9732 ∼ 0.5273	0.5296 ∼ 2.1728	2.1749 ∼ 4.3006	
Age, year (SD)	50.26(9.80)	49.54(9.32)	49.80(9.86)	51.45(10.10)	< 0.001
Sex (%)					0.270
Male	1,922(46.08)	723(52.51)	761(53.71)	765(55.56)	
Female	2,249(53.92)	654(47.49)	656(46.29)	612(44.44)	
Race (%)					< 0.001
Han	245(5.87)	92(6.68)	93(6.56)	60(4.36)	
Hui	1,393(33.40)	418(30.36)	470(33.17)	505(36.67)	
Uighur	1,395(33.45)	605(43.94)	400(28.23)	390(28.32)	
Kazakh	1,108(26.56)	247(17.94)	442(31.19)	419(30.43)	
Others	30(0.72)	15(1.09)	12(0.85)	3(0.22)	
BMI, kg/m² (SD)	26.52(4.32)	26.38(4.19)	26.54(4.61)	26.63(4.12)	0.302
Education level (%)					< 0.001
Elementary school and below	2,985(71.57)	913(66.30)	1,012(71.42)	1,060(76.98)	
Middle School	889(21.31)	342(24.84)	303(21.38)	244(17.72)	
High School and above	297(7.12)	122(8.86)	102(7.20)	73(5.30)	
Marriage (%)					0.807
Married	3,698(88.66)	1,219(88.53)	1,252(88.36)	1,227(89.11)	
Single/divorced	473(11.34)	158(11.47)	165(11.64)	150(10.89)	
Alcohol consumption (%)					< 0.001
Never	3,492(83.72)	1,124(81.63)	1,165(82.22)	1,203(87.36)	
Occasionally	643(15.42)	237(17.21)	238(16.80)	168(12.20)	
Frequently	36(0.86)	16(1.16)	14(0.99)	6(0.44)	
Smoking status (%)					< 0.001
Never	3,143(75.35)	995(72.26)	1,061(74.88)	1,087(78.94)	
Occasionally	91(2.18)	23(1.67)	34(2.40)	34(2.47)	
Frequently	937(22.46)	359(26.07)	322(22.72)	256(18.59)	
Physical activity (%)					< 0.001
Never	3,395(81.40)	1,136(82.50)	1,177(83.06)	1,082(78.58)	
Occasionally	585(14.03)	178(12.93)	167(11.79)	240(17.43)	
Frequently	191(4.58)	63(4.58)	73(5.15)	55(3.99)	
History of fractures (%)					0.958
No	3,876(92.93)	1,279(92.88)	1,319(93.08)	1,278(92.81)	
Yes	295(7.07)	98(7.12)	98(6.92)	99(7.19)	

### Nutrient intake analysis of different DII subgroups

Table 2 shows the dietary energy and nutrient levels of the study participants according to DII tertiles. Participants with higher DII scores had lower energy, protein, fat, carbohydrate, dietary fibre, cholesterol, vitamin A, vitamin B1, vitamin B2, vitamin B3, vitamin B9, vitamin C, vitamin D, vitamin E, magnesium, iron, zinc, and selenium intake; however, they had higher vitamin B6 and vitamin B12 intake.

**Table 2:** The dietary energy and nutrients of participants by dietary inflammatory index (DII) tertiles

Dietary energy and nutrients intake (IQR)	Tertiles of DII			*P* value
	T1	T2	T3	
Energy, kcal/day	4,515.84(6,728.50)	2,525.98(1,353.47)	1,910.14(769.34)	< 0.001
Protein, g/day	101.76(130.76)	63.27(33.34)	49.84(16.92)	< 0.001
Fat, g/day	71.37(41.47)	55.31(37.66)	51.62(29.97)	< 0.001
Carbohydrate, g/day	861.76(1,515.78)	458.29(289.20)	315.87(151.11)	< 0.001
Dietary fibre, g/day	58.53(160.51)	20.38(13.75)	9.68(6.53)	< 0.001
Cholesterol, mg/day	213.90(194.07)	115.19(185.48)	80.33(97.87)	< 0.001
Vitamin A, ug/day	3,468.73(11,051.03)	913.30(2,151.21)	217.70(407.96)	< 0.001
Vitamin B1, mg/day	1.45(3.47)	0.58(0.33)	0.34(0.21)	< 0.001
Vitamin B2, mg/day	2.04(3.77)	1.00(0.76)	0.55(0.43)	< 0.001
Vitamin B3, mg/day	26.66(38.20)	15.09(7.80)	10.54(3.77)	< 0.001
Vitamin B6, ug/day	53.09(32.01)	62.27(35.34)	63.34(34.77)	< 0.001
Vitamin B9, ug/day	165.96(67.23)	157.40(54.78)	149.08(53.59)	< 0.001
Vitamin B12, ug/day	264.09(171.64)	274.57(146.14)	284.51(137.21)	< 0.001
Vitamin C, mg/day	1,290.69(2,036.61)	619.47(518.23)	464.26(211.07)	< 0.001
Vitamin D, ug/day	2.22(5.21)	1.60(4.25)	1.09(1.08)	< 0.001
Vitamin E, mg/day	45.30(30.25)	32.11(24.49)	26.95(16.35)	< 0.001
Magnesium, mg/day	564.69(1,217.51)	221.51(142.32)	117.84(72.19)	< 0.001
Iron, mg/day	55.79(118.75)	28.42(24.92)	11.25(9.89)	< 0.001
Zinc, mg/day	17.63(42.94)	6.70(5.32)	3.09(2.47)	< 0.001
Selenium, ug/day	48.46(132.46)	16.08(13.97)	7.45(6.65)	< 0.001

### The association between DII scores and osteoporosis

**Fig. 2:**
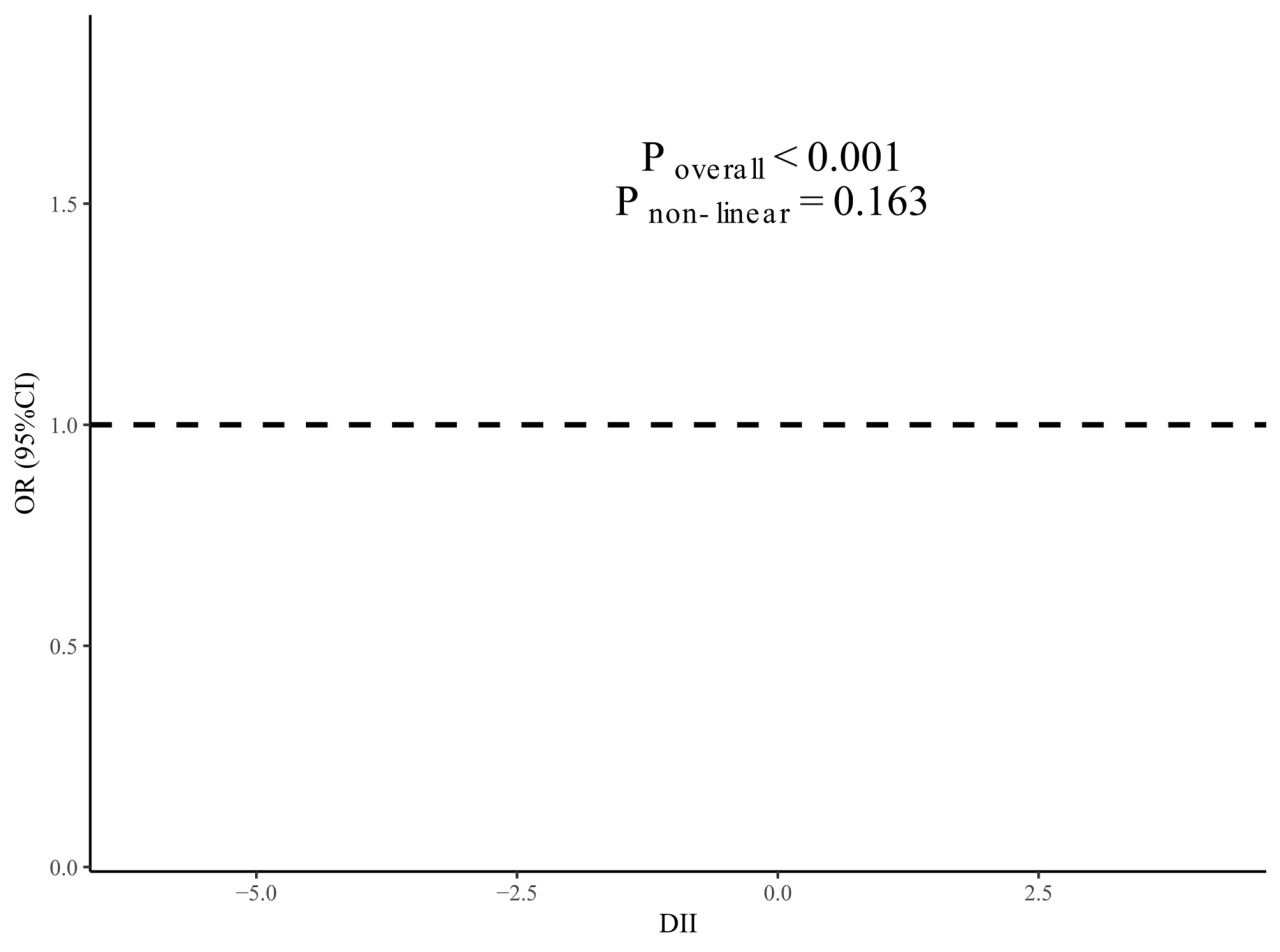
Nonlinear dose‒response curves between the dietary inflammatory index (DII) and osteoporosis incidence. We adjusted for sex, age, race, BMI, education level, alcohol consumption, smoking status, physical activity and history of fractures

The association between DII scores and osteoporosis incidence is presented in Table 3. According to the Crude model, compared with the T1 subgroup, the DII score was more strongly associated with osteoporosis risk in the T2 (*OR* = 1.59; 95% *CI* = 1.21, 2.08) and T3 (*OR* = 2.20; 95% *CI* = 1.70, 2.86) subgroups. Analysis using each one-unit increase in the DII score led to a similar result (*OR* = 1.23; 95% *CI* = 1.17, 1.30). After adjusting for relevant confounding factors (multivariate model), none of the associations changed significantly. Spline variables confirmed that there was no departure from a linear association (*P* = 0.163), and the graphical representation of the relation was consistent with a linear relation (Fig. 2).

**Table 3:** Association between the dietary inflammatory index (DII) score and osteoporosis

Model	Tertiles of DII			*P*_trend_	Each one-unit increase of DII
	T1	T2	T3		
Crude *OR* (95% *CI*)	Ref	1.59(1.21, 2.08)^2^	2.20(1.70, 2.86)^2^	< 0.001	1.23(1.17, 1.30)^2^
Multivariate *OR* (95% *CI*)^1^	Ref	1.42(1.08, 1.87)^2^	1.87(1.44, 2.45)^2^	< 0.001	1.19(1.13, 1.26)^2^

^1^Adjusted for sex, age, race, BMI, education level, alcohol consumption, smoking status, physical activity and history of fractures. ^2^*P* value < 0.05

**Fig. 3:**
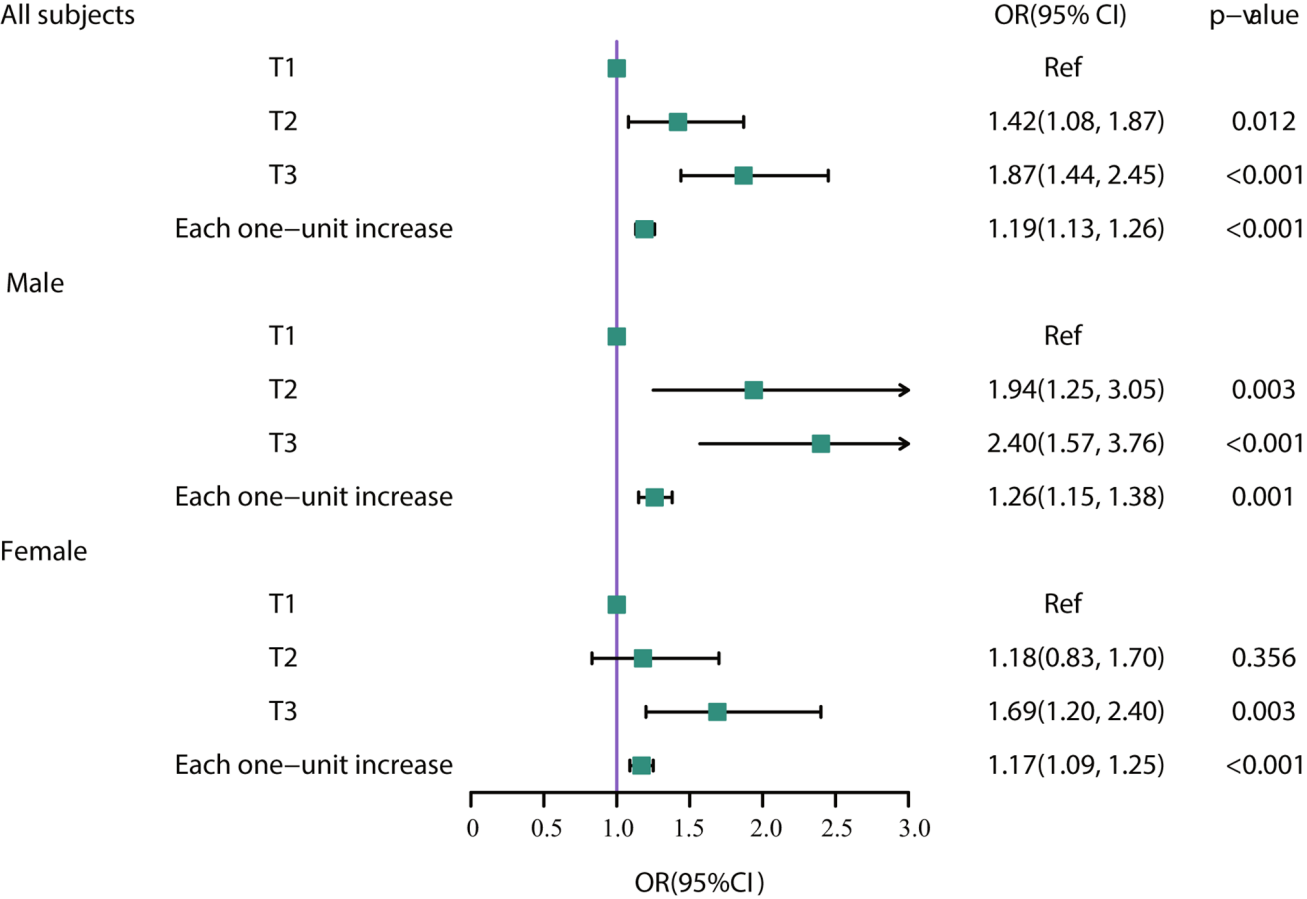
Associations between the dietary inflammatory index (DII) and osteoporosis stratified by sex. Adjusted for age, race, BMI, education level, alcohol consumption, smoking status, physical activity and history of fractures

Figure 3 shows the association between DII and osteoporosis stratified by sex. In females, an increased risk of osteoporosis was found only in the DII in the T3 group compared to the T1 group, but in males, both the T2 and T3 groups had an increased risk of osteoporosis compared to the T1 group. When DII was analysed as a continuous variable, a higher DII was also found to lead to a greater risk of osteoporosis in males than in females.

### The association between inflammatory factors and osteoporosis

Table 4 presents the relationships between 13 serum inflammatory factors and osteoporosis. We observed that increased serum IL-1β, IFN-γ, IL-6, IL-10, IL-12p70, IL-17, and IL-23 were significantly positively associated with an elevated risk of osteoporosis, and IL-6, IL-10, IL-12p70, IL-17, and IL-23 were positively linked to an increased risk of osteoporosis after adjusting for confounders (all *P* < 0.05).

**Table 4:** Associations between serum inflammatory factor concentrations and osteoporosis

Inflammatory factor	Crude model		Multivariate model^1^	
	OR (95% CI)	*P* value	OR (95% CI)	*P* value
IL-1β	1.04(1.01, 1.08)	0.024^2^	1.04(1.00, 1.08)	0.061
IFN-α2	1.03(0.84, 1.22)	0.731	1.06(0.82, 1.29)	0.602
IFN-γ	1.06(1.00, 1.12)	0.034^2^	1.05(0.99, 1.13)	0.125
TNF-α	1.00(0.97, 1.01)	0.655	1.00(0.98, 1.02)	0.850
MCP-1	1.00(1.00, 1.00)	0.193	1.00(1.00, 1.00)	0.175
IL-6	1.06(1.02, 1.12)	0.012^2^	1.06(1.02, 1.13)	0.034^2^
IL-8	1.01(0.99, 1.03)	0.137	1.04(1.00, 1.08)	0.034
IL-10	1.08(1.03, 1.15)	0.004^2^	1.10(1.04, 1.19)	0.005^2^
IL-12p70	1.20(1.07, 1.42)	0.011^2^	1.24(1.06, 1.50)	0.016^2^
IL-17	1.24(1.08, 1.47)	0.008^2^	1.27(1.08, 1.56)	0.012^2^
IL-18	1.00(0.98, 1.01)	0.762	1.00(0.98, 1.01)	0.833
IL-23	1.03(1.01, 1.06)	0.016^2^	1.03(1.01, 1.07)	0.032^2^
IL-33	1.00(0.98, 1.01)	0.897	1.01(0.98, 1.03)	0.424

^1^Adjusted for sex, age, race, BMI, education level, alcohol consumption, smoking status, physical activity and history of fractures. ^2^*P* value < 0.05

### Sensitivity analysis

According to the sensitivity analysis, a higher energy-adjusted DII score was positively associated with osteoporosis risk (*P*_trend_=0.008, Table S1), and elevated levels of IL-6, IL-10, IL-12p70, IL-17, and IL-23 were also associated with increased osteoporosis risk (*P* < 0.05, Table S2). In addition, elevated IL-8 concentrations were found to be associated with a greater risk of osteoporosis (*P* < 0.05, Table S1).

## Discussion

Data of 4,171 study participants were analysed in this study, of which the detection rate of osteoporosis was 10.48%, which is lower than the 13.4% prevalence of osteoporosis in adults over 50 years of age shown in a large multicentre study in China [23] and lower than the global prevalence of osteoporosis of 19.7% [24]. This may be because the population of this study was predominantly aged 50 years and younger, accounting for 54.93% of the total population.

Diet plays a key role in the regulation of inflammation, and there is growing evidence that certain foods, food components and nutrients can modulate the inflammatory state of the body [25]. In a study of a US population with chronic kidney disease, the DII ranged from − 5.41 ∼ 4.23, and the overall diet was anti-inflammatory [26]. In a study of pregnant women in Beijing, China, the DII ranged from − 76.05 ∼ 246.68, suggesting that the DII varies widely among individuals [27]. In the subcohort of the China Health and Nutrition Survey (CHNS), the median DII score of the study population was 0.64 (IQR − 1.74–1.46) [28]. Previous studies have focused on special populations or on European and American countries and non-multiethnic regions in China; however, different dietary structures in different geographic regions and among different ethnic groups have led to differences in findings, and few studies have focused on the inflammatory potential of the diets of multiethnic populations in Xinjiang, China. In the present study, DII scores were predominantly positive, and the diets were proinflammatory. This may be due to the site selection of this study in Huocheng County, Ili, Xinjiang, which is an area inhabited by ethnic minorities, where proinflammatory foods such as pasta, beef and mutton, and dairy products are mostly consumed, and the intake of anti-inflammatory foods such as vegetables and fruits is relatively low. The present study compared nutrient intake among the DII subgroups and revealed that as the DII score increased, the participants consumed less energy, protein, fat, carbohydrate, dietary fibre, cholesterol, vitamin A, vitamin B1, vitamin B2, vitamin B3, vitamin B9, vitamin C, vitamin D, vitamin E, magnesium, iron, zinc, and selenium; however, studies have indicated that nutrients such as dietary fibre, vitamin C, and vitamin E have anti-inflammatory effects and can reduce the level of inflammation in the body [29, 30]. Conversely, as DII scores increased, both vitamin B6 and vitamin B12 intake increased in participants, which is consistent with other studies that have shown an association between vitamin B12 intake and the DII score [17].

Inflammatory responses play an important role in the development of osteoporosis [8–11], and diet should not be overlooked as a potential source of inflammation in daily life [31]. Our study revealed a positive association between DII scores and the risk of osteoporosis, suggesting that greater dietary-induced inflammation may increase the risk of osteoporosis. Our findings on the association between DII scores and the risk of osteoporosis are consistent with several earlier studies that reported increased odds of osteoporosis due to proinflammatory diets [32, 33]. In addition, a higher intake of cholesterol-rich foods can also cause an increase in cholesterol levels in the body, and studies have shown that cholesterol in the body plays an important role in bone metabolism, which is positively correlated with a higher BMD [34]. The saturated fatty acids in processed meat may cause calciuria and have adverse effects on osteoblast formation or bone mineralization [35]. Conversely, diets rich in fruits, vegetables, and dairy products have a lower risk of osteoporosis than diets rich in meat [36, 37], and such diets with anti-inflammatory properties have greater antioxidant capacity to reduce oxidative stress [14, 38, 39], which can increase bone remodelling, improve bone repair and reduce bone loss [40]. When stratified by sex, a slightly stronger association was found for males than for females in the current study. Inconsistent with our study, the DII score was found to be positively associated with the risk of osteoporosis in women in a study of a US population with chronic kidney disease [26]. However, this association was not apparent in a male chronic kidney disease population, possibly because the study population included chronic kidney disease patients, who tend to require more high-quality protein supplements for high-quality proteolytic metabolism, and the intake of proinflammatory diets containing high-quality proteins promotes protein synthesis and metabolism in males compared to females [41]. Inconsistent with the results of our study, in a Korean study [32], higher DII scores were associated with an increased risk of osteoporosis in women, while no association was found in men. This may be due to differences in dietary structure and ethnicity in different geographic regions, leading to differences in the study results. This difference between the sexes can be explained by the effects of oestrogen, which promotes osteoblastogenesis, differentiation, and proliferation, but oestrogen deficiency can cause accelerated bone loss, leading to osteoporosis [42]. The number of young women in our study was relatively high, so the association between DII scores and the risk of osteoporosis was weaker in the female group than in the male group. In addition, sex differences in food choices could be used to explain this result. Males have been reported to be more reluctant to follow healthy dietary recommendations than females [43, 44]. However, further studies are needed to replicate these findings.

The mechanism underlying the association between a proinflammatory diet and osteoporosis may be related to the expression and secretion of inflammatory factors; a proinflammatory diet can cause disorders in the body’s immune system and promote the development of inflammatory responses and increased production of proinflammatory cytokines such as IL-1 and TNF-α [45]. Inflammatory factors such as IL-1 can directly promote bone loss by stimulating osteoclast formation and maturation and can also indirectly promote RANKL expression to enhance osteoclast-mediated bone resorption [46, 47]. The results of the present study showed that the serum IL-6, IL-17 and IL-23 levels were positively correlated with the risk of osteoporosis, in agreement with previous studies. Experimental evidence suggests that IL-6 is a pathogen that induces bone loss in several skeletal-related diseases [48] and that IL-6 stimulates the NF-κB signalling pathway, which activates downstream inflammatory cytokine levels. Thus, this vicious cycle of NF-κB signalling promotes the survival and differentiation of osteoclasts and is detrimental to bone health [49]. IL-17 is a proinflammatory cytokine secreted mainly by activated T cells that enhances osteoclast formation by inducing autophagy in bone marrow macrophages and negatively affects osteoblasts by affecting mineralization and ALP activity, which in turn affects the development of osteoporosis [50, 51]. In addition, IL-23 can assist in the onset of bone destruction via IL-17, which further amplifies TH17 cell differentiation, and fully differentiated TH17 cells can express IL-17 and induce osteoclastogenesis [52, 53], perpetuating the osteolytic effect and further aggravating osteoporosis. IL-10 is an immunosuppressive cytokine with a significant inhibitory effect on elevated levels of inflammatory cytokines, which inhibits the differentiation of osteoclasts by suppressing NFATc1 activity and Ca^2+^ mobilization and induces the differentiation of osteoblasts by downregulating microRNA 7025-5p [54, 55]. Similarly, the expression of IL-12 plays a key role in inflammatory bone resorption [56]. It has been shown that IL-12 can inhibit osteoclastogenesis by reducing NFATc1 expression [57], while animal experiments have demonstrated that IL-12 can inhibit IFN-γ-induced osteoclast formation in a T lymphocyte-deficient mouse model [58] and play a protective role during bone loss. However, in the present study, increased levels of IL-10 and IL-12p70 were positively correlated with the risk of osteoporosis, possibly because after suffering from osteoporosis, the body mobilizes relevant organs, tissues or cells to replace or compensate for their metabolism and function so that a new balance is established in the body. IL-10 and IL-12p70 are able to reduce bone loss to some extent [54, 55, 59]; therefore, IL-10 and IL-12p70 production may increase to compensate for this decrease. The exact cause needs to be further investigated.

Although previous studies have focused on the relationship between proinflammatory diets or specific nutrients and the risk of osteoporosis, the present study analysed for the first time the relationships of the DII score and inflammatory factors with osteoporosis in the multiethnic region of Xinjiang, China, which fills the research gap regarding multiethnic regions and provides a basis for the potential inflammatory response mechanisms between proinflammatory diets and osteoporosis. Second, the DII scores in this study were calculated according to the methods of Shivappa et al. [17], which enhanced comparability between studies. In addition, the results of the sensitivity analysis after adjusting for energy intake confirmed the stability of the study results. This study has some limitations because it was a cross-sectional study, and it is not clear whether people with osteoporosis have a preference for proinflammatory diets or whether proinflammatory diets help to promote or maintain osteoporosis; In addition, pharmacologic factors such as alendronate [60] and Denosumab [61, 62], which affect osteoporosis, and genetic factors [63] were not considered in the study. Therefore, the association between DII scores and the risk of osteoporosis still needs to be confirmed in a prospective analysis.

## Conclusions

In conclusion, diets with greater inflammatory potential were significantly associated with an increased risk of osteoporosis, while inflammatory factors were also associated with a greater risk of osteoporosis. Therefore, it is recommended that bone health be maintained by consuming more anti-inflammatory foods, such as fruits and vegetables. Future studies, such as prospective studies, can be conducted to determine the relationship between dietary inflammatory effects and osteoporosis and provide a scientific basis for further research on the inflammatory mechanisms of osteoporosis.

### Electronic supplementary material

Below is the link to the electronic supplementary material.

Supplementary Material 1

## Data Availability

No datasets were generated or analysed during the current study.

## Abbreviations

DII: Dietary Inflammatory Index
QUS: Quantitative ultrasound
