# Supplementary material for: Associations of dietary inflammatory index scores and serum inflammatory factors with the risk of osteoporosis: a cross-sectional study from Xinjiang, China
Source: J Orthop Surg Res. 2024 Jul 23;19:423. doi: 10.1186/s13018-024-04866-x (PMC11264401; doi:10.1186/s13018-024-04866-x)
Supplement: Supplementary file 1 — Supplementary Material 1 [file 13018_2024_4866_MOESM1_ESM.docx]

**Supplementary Information**

| **Table S1.** Association of dietary inflammatory index (DII) and osteoporosis after adjustment of energy intake. | | | | | |
| --- | --- | --- | --- | --- | --- |
| **Model** | **Tertiles of DII** | | | ***P*_trend_** | **Each one-unit increase of DII** |
|  | **T1** | **T2** | **T3** |  |  |
| Osteoporosis *OR*(95% *CI*) | Ref | 1.13(0.85, 1.53) | 1.39(1.02, 1.89)^1^ | 0.008 | 1.15(1.08, 1.24)^1^ |
| Adjusted sex, age, race, BMI, education level, alcohol consumption, smoking status, physical activity, history of fractures and energy intake. *^1^P* value<0.05. | | | | | |

| **Table S2.** Association of serum inflammatory factor concentration and osteoporosis after adjustment of energy intake. | | |
| --- | --- | --- |
| **Inflammatory factor** | **Osteoporosis** | |
|  | ***OR* (95% *CI*)** | ***P* value** |
| IL-1β | 1.04(1.01, 1.08) | 0.024^1^ |
| IFN-α2 | 1.05(0.81, 1.28) | 0.676 |
| IFN-γ | 1.05(0.99, 1.13) | 0.136 |
| TNF-α | 1.00(0.97, 1.02) | 0.964 |
| MCP-1 | 1.00(1.00, 1.00) | 0.145 |
| IL-6 | 1.06(1.01, 1.13) | 0.037^1^ |
| IL-8 | 1.04(1.00, 1.08) | 0.040^1^ |
| IL-10 | 1.11(1.04, 1.19) | 0.005^1^ |
| IL-12p70 | 1.24(1.06, 1.50) | 0.017^1^ |
| IL-17 | 1.27(1.08, 1.55) | 0.013^1^ |
| IL-18 | 1.00(0.98, 1.01) | 0.797 |
| IL-23 | 1.03(1.01, 1.07) | 0.038^1^ |
| IL-33 | 1.01(0.98, 1.02) | 0.464 |
| Adjusted sex, age, race, BMI, education level, alcohol consumption, smoking status, physical activity, history of fractures and energy intake.. ^1^*P* value<0.05. | | |
